# CD44v6 chimeric antigen receptor T cell specificity towards AML with FLT3 or DNMT3A mutations

**DOI:** 10.1002/ctm2.1043

**Published:** 2022-09-26

**Authors:** Ling Tang, Hongming Huang, Yutong Tang, Qing Li, Jue Wang, Dengju Li, Zhaodong Zhong, Ping Zou, Yong You, Yang Cao, Yingjie Kong, Anyuan Guo, Shu Zhou, Huimin Li, Fankai Meng, Yi Xiao, Xiaojian Zhu

**Affiliations:** ^1^ Institute of Hematology Union Hospital, Tongji Medical College Huazhong University of Science and Technology Wuhan China; ^2^ Department of Hematology Affiliated Hospital of Nantong University Nantong Jiangsu China; ^3^ Department of Hematology, Wuhan No. 1 Hospital, Tongji Medical College Huazhong University of Science and Technology Wuhan China; ^4^ Department of Hematology Tongji Hospital, Tongji Medical College Huazhong University of Science and Technology Wuhan China; ^5^ Key Laboratory of Molecular Biophysics of the Ministry of Education College of Life Science and Technology Huazhong University of Science and Technology Wuhan China; ^6^ Department of Hematology Zhongnan Hospital Affiliated to Wuhan University Wuhan China

**Keywords:** CD44 isoform 6, chimeric antigen receptor T‐cell, acute myeloid leukaemia, FMS‐like tyrosine kinase 3 mutation, DNA methyltransferase 3A mutation, methylation

## Abstract

**Background:**

Chimeric antigen receptor T‐cell (CAR‐T) therapy for acute myeloid leukaemia (AML) has thus far been elusive, in part due to target restriction and phenotypic heterogeneity of AML cells. Mutations of the FMS‐like tyrosine kinase 3 (*FLT3*) and DNA methyltransferase 3A (*DNMT3A*) genes are common driver mutations that present with a poor prognosis in AML patients. We found that AML patients with *FLT3* or *DNMT3A* mutations had higher expression of CD44 isoform 6 (CD44v6) compared to normal specimens. Therefore, we intended to demonstrate CD44v6 could be a specific option for AML with *FLT3* or *DNMT3A* mutations.

**Methods:**

Internal tandem duplication (ITD) mutations of *FLT3* (*FLT3*/ITD) knock‐in clone and *DNMT3A*‐R882H mutant clones of SKM‐1 cells were generated using CRISPR/Cas9 and lentiviral transfection, respectively. CD44v6 CAR‐T cells were constructed by transfecting T cells with lentivirus containing CD44v6 CAR. CD44v6 expression in AML cell lines, AML patients and healthy donors was evaluated by flow cytometry. DNA methylation assays were used to analyse the mechanisms of *FLT3* and *DNMT3A* mutations affecting CD44v6 expression.

**Results:**

Aberrant overexpression of CD44v6 was observed in AML cell lines with *FLT3* or *DNMT3A* mutations compared to the wild‐type SKM‐1 or K562 cells. AML patients with *FLT3* or *DNMT3A* mutations had higher expression of CD44v6 compared to normal specimens. Then we constructed CD44v6 CAR‐T cells and found that CD44v6 CAR‐T specifically lysed CD44v6^+^ cells, accompanied by cytokines release. No significant killing effect was observed from CD44v6^‐^ AML cells and normal cells after co‐culture with CD44v6 CAR‐T. These results were also observed in vivo. Furthermore, we found that *FLT3* or *DNMT3A* mutations induced CD44v6 overexpression by downregulating the CpG methylation of CD44 promoter.

**Conclusions:**

Collectively, CD44v6 is a promising target of CAR‐T for AML patients with *FLT3* or *DNMT3A* mutations.

## Graphical abstract

1. CD44v6 is an ideal antigen of CAR‐T therapy for AML with *FLT3* or *DNMT3A* mutations.
2. CD44v6 CAR‐T cells displayed strong anti‐leukemic effects and safety in vitro and in vivo.
3. *FLT3* or *DNMT3A* mutations induced CD44v6 overexpression by downregulating the methylation of CD44 promoter.

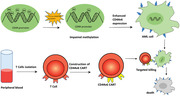

## 1 INTRODUCTION

The disease acute myeloid leukaemia (AML) is heterogeneous, and the majority of patients would suffer a relapse after undergoing standard induction chemotherapy.^1^, ^2^ In order to stratify AML patients based on their risk and guide clinicians toward the most effective treatment, molecular and cytogenetic profiling are becoming increasingly common. Genomic data suggest that mutational, clonal, and epigenetic heterogeneity contribute to AML's biological complexity.^3^, ^4^ In AML, the frequency of FMS‐like tyrosine kinase 3 (*FLT3*) and DNA methyltransferase 3A (*DNMT3A*) mutations was approximately 37% and 23%, respectively.^5^ *FLT3* or *DNMT3A* mutant AML patients presented with lower remission rates, higher recurrence rates, and poorer overall survival. Moreover, studies have reported that *FLT3* mutations often co‐occur with *DNMT3A* mutations, leading to a worse prognosis.^6^ Internal tandem duplication (ITD) mutations of *FLT3* are among the most frequent genetic alterations in AML.^7^ FLT3 inhibitors improve remission rates of AML patients, but drug toxicity and acquired resistance remain significant challenges.^8^ Although allogeneic hematopoietic stem cell transplantation (allo‐HSCT) presents a high cure rate for AML, most patients cannot receive and tolerate the transplant. Relapse following allo‐HSCT remains a major challenge and is associated with worse prognosis^9^. Currently, there are no available inhibitors targeting mutations such as *DNMT3A*. Over the years, chimeric antigen receptor T cell (CAR‐T) therapy for AML has been developed. However, the treatment of AML with single‐specific CARs redirecting T cells against CD33^10^, CD123^11^, CLL‐1^12^, FLT3^13^ and NKG2D^14^ is hindered by the fact that these antigens are expressed not only on AML blasts, but also on hematopoietic stem cells (HSCs), progenitor and mature hematopoietic cells of the myeloid lineage and endothelial cells. Therefore, this strategy of CAR‐T in AML effectively requires more leukaemia‐specific antigens.

CD44 is selectively and highly expressed in hematopoietic and epithelial tumours, and is considered to be an indication of cancer stem cells for multiple cancers.^15^ The isoform 6 of CD44,^16^ named CD44v6, is relatively tumour‐limited and associated with poor prognosis in AML.^17^, ^18^, ^19^ In AML, CD44v6 was selectively expressed in primary cells of AML but not in HSCs, thus ensuring its safety as a CAR‐T therapeutic antigen.^18^, ^20^ During this I/IIa phase of clinical trial gov NCT04097301, CD44v6 CAR‐T cells were injected into AML patients which proved them to be effective and safe. No further clinical data was present currently. Besides, there is no data indicating the association between CD44v6 and molecular genetic abnormalities. In this study, we have generated a CD44v6 CAR‐T and demonstrated advantages of CD44v6 CAR‐T cells in AML with *FLT3* or *DNMT3A* mutations.

## 2 MATERIALS AND METHODS

### 2.1 Construction of *FLT3*/ITD mutant cell line

The SKM‐1‐FLT3 cells were donated by Prof. Jue Wang. SKM‐1 cells knocked into a 21‐bp ITD fragment by CRISPR/Cas9 system were named SKM‐1‐FLT3 cells. Successful knock‐in of the *FLT3*/ITD mutation was confirmed by increased FLT3 expression and phosphorylation of classical FLT3 downstream signalling. These results have been described previously.^21^

### 2.2 Construction of *DNMT3A*‐R882H mutant cell line

The *DNMT3A* mutant SKM‐1 cells were donated by Prof. Dengju Li. For detailed construction methods and related data, please refer to previous research.^22^ The brief process was as follows: a lentiviral vector pCDH‐EF1‐MCS‐T2A‐copGFP was used to clone the full‐length human *DNMT3A* cDNA. Mutant strain of *DNMT3A*‐R882H (CGC > CAC) was identified by DNA sequence. After 72 h, the supernatant with infectious lentivirus was gathered and identified by PCR and sequencing to verify the overexpression of the target gene.

SKM‐1 cells infected with the overexpressed lentivirus were separated into single cells, and the grown monoclonal cells were expanded and cultured. The target fragment was amplified by PCR and Sanger. Finally, the two mutant clones were named SKM‐1‐DNMT3A‐SC2 and SKM‐1‐DNMT3A‐SC3, respectively.

### 2.3 CD44v6 expression in target cells

When the target cells were cell lines or T cells, incubation of the cells for 30 min was done with CD44v6‐PE (BD Bioscience, USA) or isotype control mouse IgG1‐PE (BD Bioscience, USA). When the target cells were peripheral blood mononuclear cells (PBMCs) from AML patients or healthy donors, the PBMCs were incubated with the following antibodies (BD Bioscience, USA): Fixable Viability Stain 510, CD117‐PE‐CY7, CD34‐APC, CD45‐APC‐CY7, HLA‐DR‐BV421, CD44v6‐PE or mouse IgG1‐PE. The samples were all detected by BD LSRFortessa X‐20 flow cytometry and analysed by FlowJo software.

### 2.4 Construction of CD44v6 CAR

The single‐chain variable fragment (scFv) sequence of the CD44v6 CAR derived from the humanized mAb bivatuzumab (BIWA‐8).^23^ CAR lentivirus containing anti‐CD44v6‐scFv‐CD8‐4‐1BB‐CD3ζ were constructed by Shanghai GeneChem (China).

### 2.5 Cell isolation, T‐cell culture and transduction

CD3/CD28 beads ((Miltenyi Biotech, Germany) were added to healthy donor CD3^+^ T cells for 48 h to activate the T cells, after which these T cells and CAR lentivirus were mixed and added into 96‐well‐plates precoated with 6 μg/cm^2^ Retronectin (Takara Bio, Japan). The multiplicity of infection was three. After centrifugation at 800 × *g* for 30 min at 32°C, the mixtures were incubated at 37°C for 24 h. The transfections were terminated by replacement of fresh X‐VIVO 15 medium with supplementation of 5% NTC serum and 200 U/ml recombinant human IL‐2 (PeproTech, USA).

### 2.6 Characterization of CAR‐T cells

Biotinylated protein L (GenScript, USA) was used to detect CAR‐expressing T cells. CAR‐T cells were incubated with biotinylated Protein L for 45 min and then washed three times with wash buffer, followed by streptavidin‐FITC (BD Bioscience, USA) for 15 min. For T cell phenotype analysis, the following antibodies were used (BD Bioscience, USA): CD4‐PerCP‐Cy5.5, CD8‐FITC, CCR7‐APC and CD45RA‐PE.

### 2.7 Cytotoxicity assay

Different effector cells:target cells (E:T) ratios of 0:1, 1:1 and 10:1 were used when co‐culturing CD44v6 CAR‐T cells or non‐transduced T (NT) cells with target cells that had stained with CFSE (BD Bioscience, USA) for 24 h. PI (BD Pharmingen, USA) was used to identify dead target cells. The cytotoxicity was assessed by the percentage of dead cells in target cells.

### 2.8 Degranulation assay

An E:T ratio of 1:1 was used when co‐culturing CD44v6 CAR‐T (or NT) cells with target cells in complete RPMI 1640 media containing anti‐CD107a‐FITC (Biolegend, USA) and protein transport inhibitor (BD Bioscience, USA) for 4 h. CD3‐APC/Cy7(Biolegend, USA), CD4‐APC (Biolegend, USA) and CD8‐BV605 (BD Bioscience, USA) were used to stain the harvested cells.

### 2.9 Cytokine release assay

An E:T ratio of 1:1 was used when co‐culturing CD44v6 CAR‐T (or NT) cells with target cells in complete RPMI 1640 media for 24 h, after which the cytokines of supernatants were measured using the BD cytometric bead array (CBA).

### 2.10 Animal experiments

All animal experiments were approved by the Institutional Animal Care and Use Committee of Tongji Medical College, Huazhong University of Science and Technology, and strictly followed the animal regulations of Hubei Province.

Five‐week‐old NOD/Shi‐scid IL2rγnull (NOG) mice, four‐week‐old BALB/c‐nu mice and BALB/c mice were all male and purchased from Vital River Laboratory Animal Technology (Beijing, China). We constructed Luc^+^ SKM‐1, Luc^+^ SKM‐1 FLT3 and Luc^+^ MV4‐11 cells expressing the firefly luciferase gene by lentiviral transfection.

BALB/c‐nu mice were subcutaneously transplanted with 1 × 10^7^ Luc^+^ SKM‐1 or Luc^+^ MV4‐11 on the left side of the groin at day 0. The mice injected with Luc^+^ MV4‐11 cells were randomly separated into three groups: one group with 1 × 10^7^ CD44v6 CAR‐T cells, one group with 1 × 10^7^ non‐transduced T (NT) cells and another group with equal volume of 200 μl phosphate buffered saline (PBS) injected into the tumour on days 8 and 15. Tumour burden was analysed by bioluminescent imaging (BLI) on days 7, 14, 21 and 28. We used the Luc^+^ SKM‐1 mice as negative controls as wild‐type SKM‐1 cells lacked CD44v6 expression. Since tumour formation of SKM‐1 cells was slower than that of MV4‐11 cells, SKM‐1 cells took longer to grow to the same tumour size as MV4‐11 cells. Therefore, the mice injected with Luc^+^ SKM‐1 cells were treated with intratumour injection of 1 × 10^7^ CD44v6 CAR‐T cells, NT cells or 200 μl PBS on days 22 and 29, and the burden was analysed by BLI on days 21, 28, 35 and 42.

NOG mice were intravenously injected with 1 × 10^7^ Luc^+^ SKM‐1 or Luc^+^ SKM‐1‐FLT3 cells on day 0, and treated with 1 × 10^7^ CD44v6 CAR‐T cells, 1 × 10^7^ NT cells or 200 μl PBS on days 15 and 29. The leukaemia development was monitored by BLI on days 14, 28 and 42.

To evaluate the safety of CAR‐T cells in vivo, 1 × 10^7^ CD44v6 CAR‐T cells, NT cells and 200 μl PBS were intravenously injected into BALB/c mice, respectively. After 10 days, the weight change of the mice was measured, and peripheral blood was collected for blood count analysis and biochemistry analysis.

For detailed description of **DNA methylation analysis,** see Supporting Information.

### 2.11 Statistical analysis

Values were expressed as mean ± SD. Evaluate significance with GraphPad Prism by Student's *t* test, one‐way or two‐way analysis of variance (ANOVA). Comparison of CD44v6 expression in precursor cells of normal subjects and AML patients was determined by Mann–Whitney test, using GraphPad Prism. Comparison of the bioluminescence intensity of PBS, NT and CD44v6 CAR‐T groups at different time points in SKM‐1‐FLT3 mice was determined by Kruskal–Wallis test. *p* values < .05 were considered statistically significant, and “ns” indicated no significance (*p* > .05).

## 3 RESULTS

### 3.1 Expression of CD44v6 on tumour cells

**FIGURE 1:**
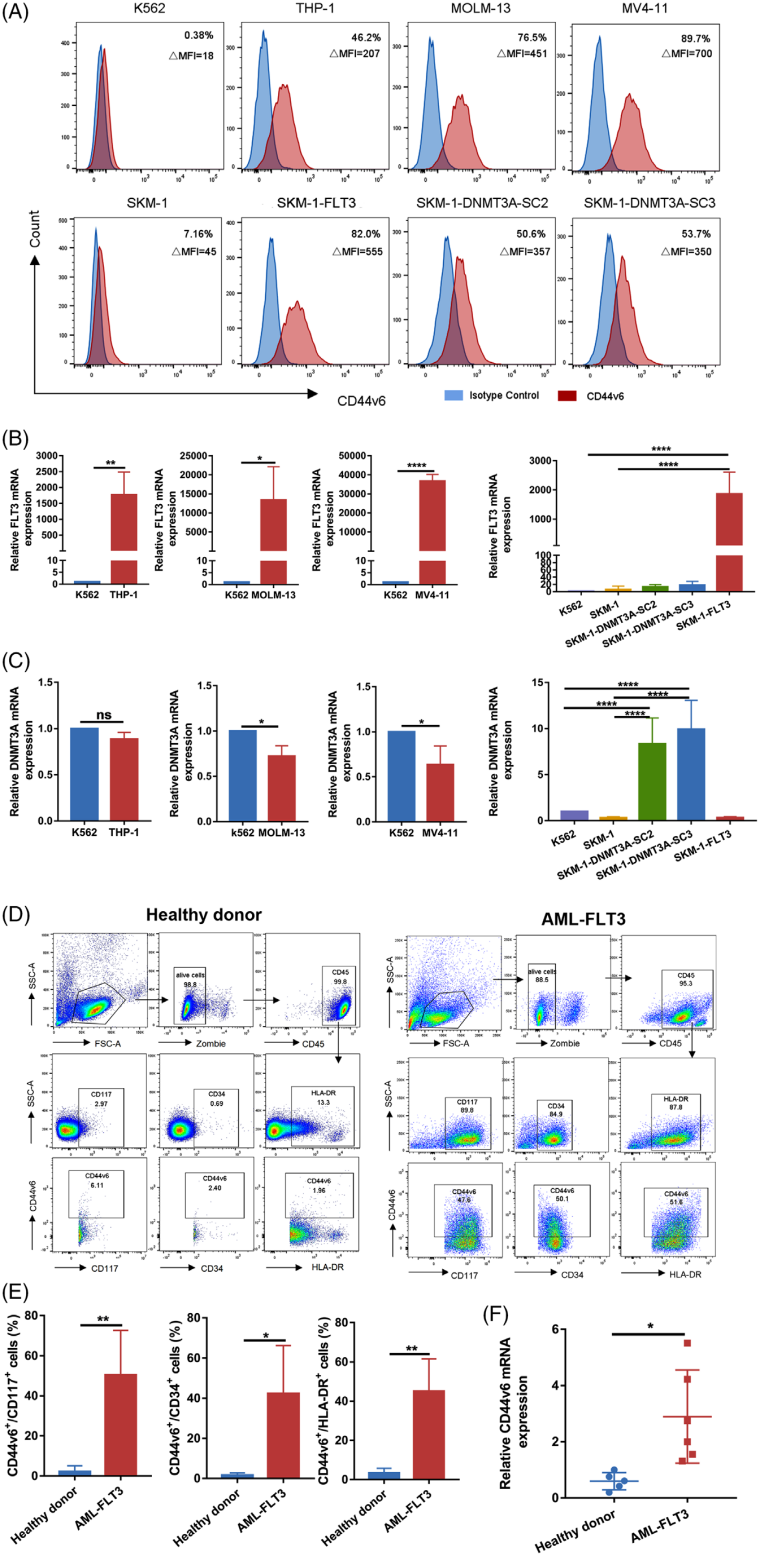
Expression of CD44v6 on tumour cells. FLT3 mutant SKM‐1 cells (SKM‐1‐FLT3) and DNMT3A mutant SKM‐1 cells (SKM‐1‐DNMT3A‐SC2 and SKM‐1‐DNMT3A‐SC3) were engineered from SKM‐1 cells. (A) The expression of CD44v6 in K562 cell line and AML cell lines THP‐1, MOLM‐13, MV4‐11, SKM‐1, SKM‐1‐FLT3, SKM‐1‐DNMT3A‐SC2 and SKM‐1‐DNMT3A‐SC3 was analysed by flow cytometry. (B) RT‐qPCR analysis of the expression of FLT3 in K562 cells (*n* = 5) and AML cell lines THP‐1 (*n* = 3), MOLM‐13 (*n* = 4), MV4‐11 (*n* = 5) (left) and SKM‐1 (*n* = 5), SKM‐1‐DNMT3A‐SC2 (*n* = 4), SKM‐1‐DNMT3A‐SC3 (*n* = 4), SKM‐1‐FLT3 (*n* = 4) (right), SKM‐1 cells were used as negative controls. (C) RT‐qPCR analysis of the expression of DNMT3A in K562 cells (*n* = 5) and AML cell lines THP‐1 (*n* = 3), MOLM‐13 (*n* = 4), MV4‐11 (*n* = 5) (left) and SKM‐1 (*n* = 5), SKM‐1‐DNMT3A‐SC2 (*n* = 5), SKM‐1‐DNMT3A‐SC3 (*n* = 5), SKM‐1‐FLT3 (*n* = 5) (right), SKM‐1 cells were used as negative controls. (D) Representative flow cytometric plots showing the percentage of CD44v6 in healthy donor (left) and FLT3 mutant AML primary (right) precursor cells (CD117^+^, CD34^+^, HLA‐DR^+^). (E) Percentage of CD44v6 in AML primary precursor cells (*n* = 7) and healthy donor cells (*n* = 6) was determined by flow cytometry, and analysed by non‐parametrical Mann–Whitney test. (F) RT‐qPCR analysis of the expression of CD44v6 in PBMCs of normal (*n* = 5) and FLT3 mutant AML (*n* = 6) *p < .05; **p < .01; ****p < .0001

Studies have reported that multiple tumour cells highly express CD44v6. We found that CD44v6 was highly expressed in AML cell lines THP‐1, MOLM‐13 and MV4‐11 and colorectal cancer cell lines SW480, Caco‐2, HT29 and HCT116, but low or not expressed in SKM‐1, A549, HepG2, and K562 cell lines (Figure 1A and Figure S1A,B).

We focused our study on AML, *FLT3*/ITD mutations occur frequently and predict a dismal prognosis^8^. FLT3 mRNA expression in the leukaemia cell lines mentioned above was detected. The results showed that THP‐1, MOLM‐13 and MV4‐11 cells highly expressed FLT3 mRNA compared to K562 cells, which was consistent with the expression of CD44v6 (Figure 1B). Studies have indicated that MOLM‐13 and MV4‐11 are *FLT3*/ITD^+^ cell lines, while THP‐1 is a *FLT3*/WT cell line.^24^, ^25^ Therefore, CD44v6 expression was associated with *FLT3* mutations or FLT3 overexpression which was also proved by our follow‐up experiments. Using the CRISPR/Cas9 system, we knocked in a 21‐bp ITD fragment in wild‐type SKM‐1 cells and named the knock‐in clone SKM‐1‐FLT3.^21^ Compared to SKM‐1 cells, SKM‐1‐FLT3 cells significantly overexpressed FLT3 mRNA and CD44v6 (Figure 1A,B).

Studies had also shown that about 25% of AML was accompanied by a genetic mutation of *DNMT3A*, the most common of which is *DNMT3A* R882H mutation.^7^, ^26^, ^27^, ^28^ We constructed *DNMT3A* mutant SKM‐1 cells by lentiviral transfection. Two mutant monoclonal R882H SC2 and R882H SC3 strains were selected and named SKM‐1‐DNMT3A‐SC2 and SKM‐1‐DNMT3A‐SC3. RT‐qPCR demonstrated that DNMT3A was highly expressed in *DNMT3A* mutated SKM‐1 cells, while absent in other cells (Figure 1C). *DNMT3A* mutant SKM‐1 cells highly expressed CD44v6 compared to SKM‐1 cells. (Figure 1A).

Then, we assayed the expression of CD44v6 in progenitor cells (CD34^+^, CD117^+^ and HLA‐DR^+^) of AML patients with *FLT3* and healthy donors by flow cytometry, and found that CD44v6 was highly expressed in AML patients but not in healthy donors (Figure 1D,E). We also measured CD44v6 mRNA expression in PBMCs from healthy donors and *FLT3* or *DNMT3A* mutant AML patients. The date revealed that AML patients with *FLT3* or *DNMT3A* (not shown) mutations overexpressed CD44v6 compared to healthy donors, which was consistent with the results of the SKM‐1 cells with *FLT3* mutation or *DNMT3A* mutation (Figure 1F).

### 3.2 Characterization of CAR‐T cells

**FIGURE 2:**
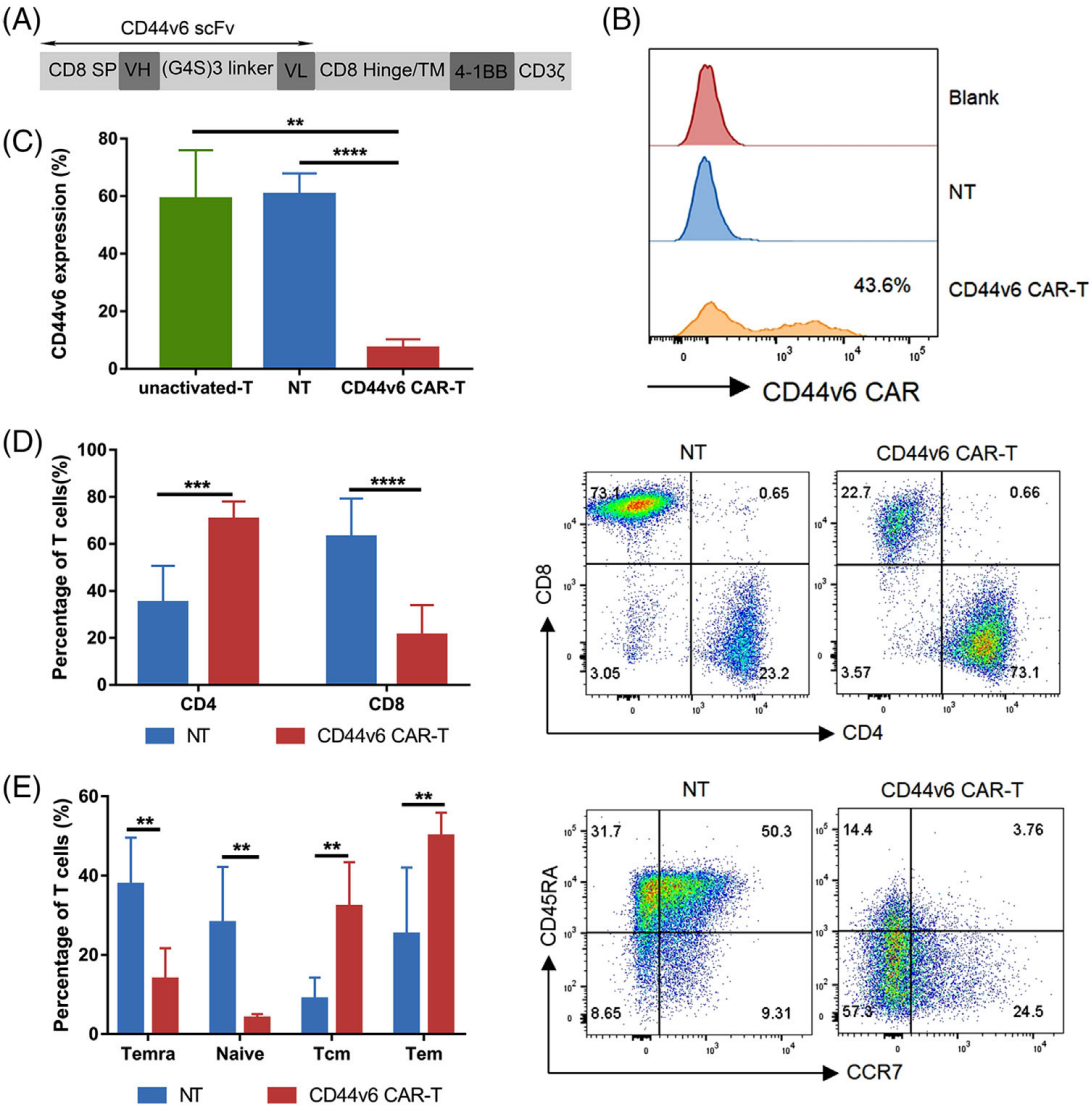
CD44v6 CAR‐T cells construction and characterization. (A) Schematic structure of the CD44v6 CAR. (B) Transduction efficiencies of primary activated T cells 7 days after transduction were analysed by flow cytometry. (C) CD44v6 expression on not activated T (unactivated‐T), non‐transduced (NT, activated) cells and CD44v6 CAR‐T cells 7 days after transduction was analysed by flow cytometry (*n* = 5). (D) The ratio of CD4^+^ and CD8^+^ T cells in NT or CD44v6 CAR‐T cells 14 days after transduction was detected by flow cytometry (*n* = 6). (E) CCR7 and CD45RA surface staining was performed on both NT and CD44v6 CAR‐T cells 14 days after transduction (*n* = 5). Tcm, central memory, CCR7^+^CD45R^−^; Tem, effector memory, CCR7^−^CD45RA^−^; Temra, terminally differentiated effector memory, CCR7^−^CD45RA^+^; Naive, CCR7^+^CD45RA^+^. Bar are depicted as the mean ± SD. ***p < .001; ****p < .0001

We constructed a CD44v6 CAR containing CD44v6 scFv, CD8α hinge and transmembrane domains, 4‐1BB costimulatory domain and CD3ζ signalling domain (Figure 2A). Seven days after transfection, the transfection efficiencies were about 40% (Figure 2B). In contrast to NT cells, CD44v6 CAR‐T cells showed decreased CD44v6 expression and CD8^+^ T cells (Figure 2C,D), but increased proportion of central memory T cells (Tcm, CD45RA^−^CCR7^+^) and effector memory T cells (Tem, CD45RA**^−^** CCR7^−^) (Figure 2E).

### 3.3 CD44v6 CAR‐T cells specifically target CD44v6^+^ tumour cell lines

**FIGURE 3:**
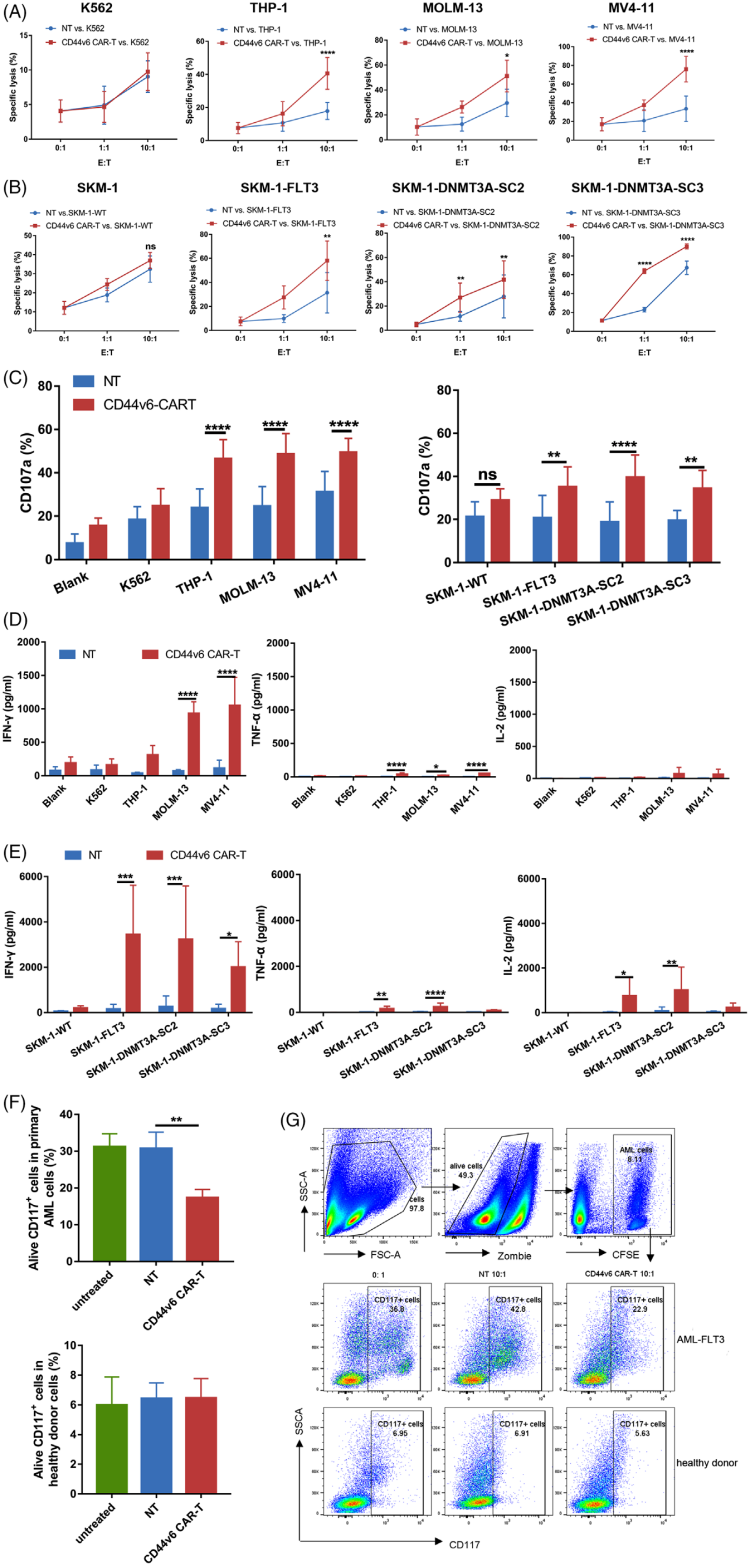
CD44v6 CAR‐T cells specifically target CD44v6^+^ tumour cells. CFSE‐labelled target cells were co‐cultured with CD44v6 CAR‐T or NT cells at the indicated E:T ratio of 0:1, 1:1 and 10:1 for 24 h. The percentage of live and dead cells was tested by flow cytometry using PI. The CFSE^+^PI^+^ cells/CFSE^+^ cells ratio was used to determine the killing rate. NT cells were used to evaluate unspecific lysis. (A) CD44v6 CAR‐T cells lysed CD44v6^+^ AML cell lines THP‐1 (*n* = 5), MOLM‐13 (*n* = 3) and MV4‐11 (*n* = 4).(B) CD44v6 CAR‐T cells lysed AML cell lines SKM‐1 (*n* = 4), SKM‐1‐FLT3 (*n* = 4) SKM‐1‐DNMT3A‐SC2 (*n* = 4) and SKM‐1‐DNMT3A‐SC3 (*n* = 3). (C) Percentage of CD3^+^CD107a^+^ T cells after being co‐cultured with target cells at an E:T ratio of 1:1 for 4 h was determined by flow cytometry (*n* = 5). Blank group was co‐cultured with complete medium, SKM‐1 cells were used as negative controls. (D) CD44v6 CAR‐T or NT cells (*n* = 4) were co‐cultured with CD44v6^+^ AML cell lines THP‐1, MOLM‐13 and MV4‐11 at an E:T ratio of 1:1 for 24 h. IFN‐γ, TNF‐α, IL‐2 amounts in the supernatants were analysed by using CBA. (E) CD44v6 CAR‐T or NT cells (*n* = 6) were co‐cultured with FLT3 or DNMT3A mutant AML cell lines SKM‐1‐FLT3, SKM‐1‐DNMT3A‐SC2 and SKM‐1‐DNMT3A‐SC3 at an E:T ratio of 1:1 for 24 h. IFN‐γ, TNF‐α and IL‐2 amounts in the supernatants were analysed by using CBA. CD44v6^−^ cell line SKM‐1 was used as a negative control. (F) PBMCs of AML samples (*n* = 4) or normal (*n* = 4) were labelled with CFSE and co‐cultured with CD44v6 CAR‐T orNT cells at an E:T of 10:1 for 24 h, and the alive CD117^+^ cells were detected by flow cytometry using Fvs and anti‐CD117. (G) Representative scatter plot of CD44v6 CAR‐T cells lysed FLT3 mutant AML and normal. Bar are depicted as the mean ± SD. *p < .05; **p < .01; ***p < .001; ****p < .0001; ns not significant

In co‐culture with leukemic cells, CD44v6 CAR‐T cells specifically lysed CD44v6^+^ tumour cells but not CD44v6^−^ SKM‐1 and K562 cells (Figure 3A,B and Figure S2A,B). The specificity and efficacy of CD44v6 CAR‐T were again confirmed when CD44v6^+^ colorectal cancer cells were used as target cells (Figure S1C). CD44v6 CAR‐T cells killing of CD44v6^+^ tumour cells were accompanied by enhanced degranulation response and cytokine release. In co‐culture with CD44v6^+^ cells, CD44v6 CAR‐T expressed a higher level of CD107a (Figure 3C) and released more cytokines such as IFN‐γ, TNF‐α (Figure 3D,E, Figure S2C,D). According to these data, CD44v6 CAR‐T specifically targeted the CD44v6 antigen.

### 3.4 CD44v6 CAR‐T cells specifically target primary AML cells

PBMCs from AML patients with *FLT3* mutations and healthy donors were used as target cells. CD44v6 was overexpressed in CD117^+^ progenitor cells of AML. Based on the proportion of CD117^+^ cells that survived after co‐culture at a 10:1 ratio, CD44v6 CAR‐T cells were shown to be specific cytotoxic against primary AML cells, but not to healthy donor cells (Figure 3F,G).

### 3.5 CD44v6 CAR‐T cells have potent anti‐tumour efficacy in vivo

**FIGURE 4:**
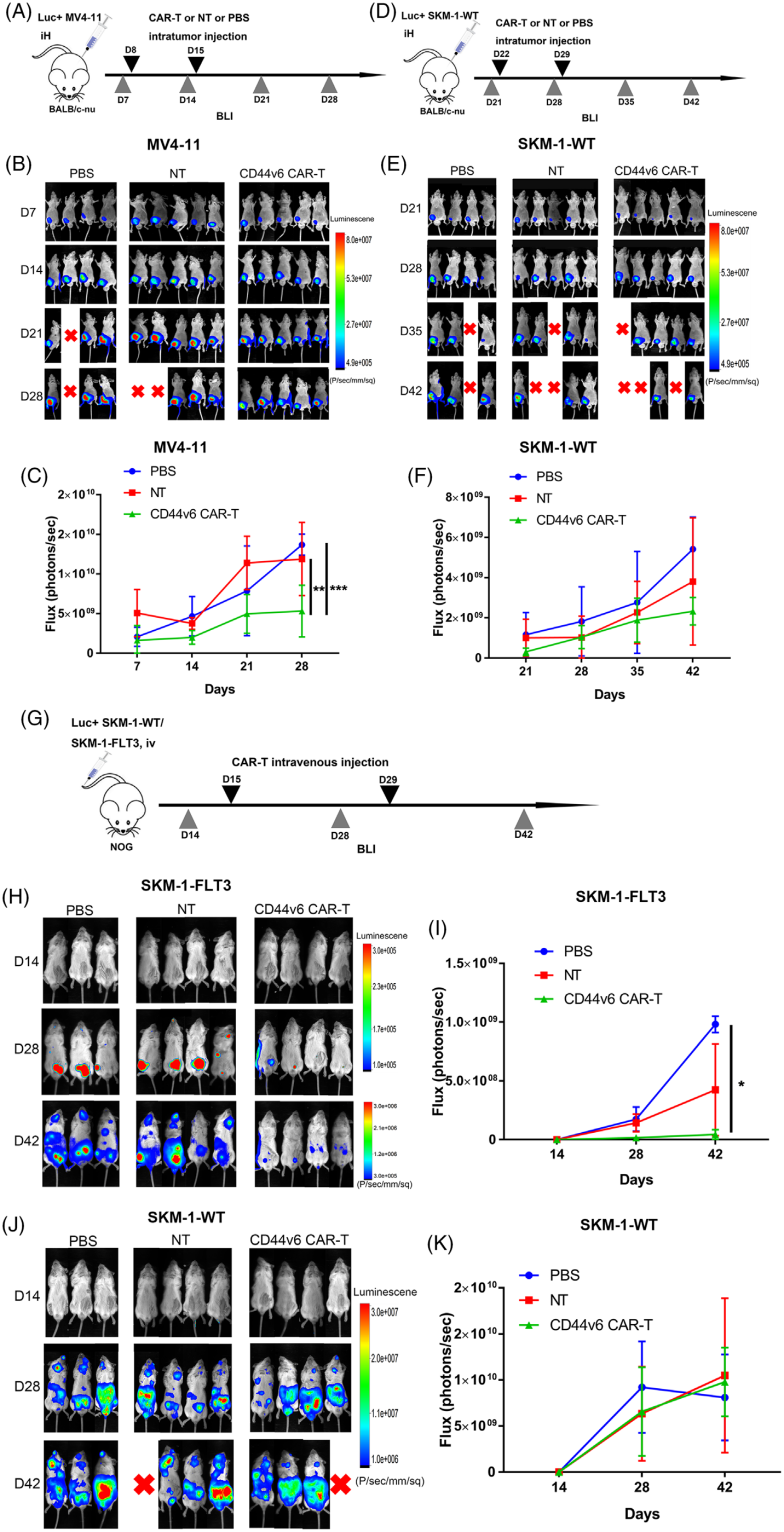
CD44v6 CAR‐T cells have potent anti‐tumour efficacy in vivo. (A) Schematic of the MV4‐11 cells xenograft model. BALB/c‐nu mice were subcutaneously injected with 1 × 10^7^ MV4‐11‐firefly luciferase (Luc^+^ MV4‐11) cells on day 0. Bioluminescent imaging (BLI) was performed on day 7 to quantify engraftment and for randomization of treatment groups. CD44v6 CAR‐T cells (1 × 10^7^) (*n* = 5), NT cells (1×10^7^) (*n* = 5), or 200 μl PBS (*n* = 4) were intratumorally injected on day 8 and day 15. Subsequently, tumour burden was analysed by BLI on days 14, 21 and 28. (B) Tumour burden of different groups of Luc^+^ MV4‐11 mice was analysed by BLI on days 7, 14, 21 and 28. (C) Bioluminescent signal for each treatment group of Luc^+^ MV4‐11 mice over time. (D) Schematic of the SKM‐1 cells xenograft model. BALB/c‐nu mice were subcutaneously injected with 1 × 10^7^ SKM‐1‐firefly luciferase (Luc^+^ SKM‐1) cells on day 0. BLI was performed on day 21 to quantify engraftment and for randomization of treatment groups. CD44v6 CAR‐T cells (1 × 10^7^) (*n* = 5), NT cells (1 × 10^7^) (*n* = 5) or 200 μl PBS (*n* = 4) were intratumorally injected on day 22 and day 29. Subsequently, tumour burden was analysed by BLI on days 28, 35 and 42. (E) Tumour burden of different groups of Luc^+^ SKM‐1 mice was analysed by BLI on days 21, 28, 35 and 42. (F) Bioluminescent signal for each treatment group of Luc^+^ SKM‐1 mice over time. (G) Schematic of the SKM‐1 or SKM‐1‐FLT3 cells xenograft model. NOG mice were intravenously injected with 1 × 10^7^ Luc^+^ SKM‐1 or Luc^+^ SKM‐1‐FLT3 cells on day 0, and treated with 1 × 10^7^ CD44v6 CAR‐T cells, 1 × 10^7^ NT cells or 200 μl PBS on days 15 and 29. The leukaemia development was monitored by BLI on days 14, 28 and 42. (H) Leukaemia development of different groups of Luc^+^ SKM‐1‐FLT3 NOG mice was analysed by BLI on days 14, 28 and 42. (I) Bioluminescent signal for each treatment group of Luc^+^ SKM‐1‐FLT3 NOG mice over time was analysed by non‐parametrical Kruskal–Wallis test. (J) Leukaemia development of different groups of Luc^+^ SKM‐1 NOG mice were analysed by BLI on days 14, 28 and 42. (K) Bioluminescent signal for each treatment group of Luc^+^ SKM‐1 NOG mice over time. Bar are depicted as the mean ± SD. *p < .05; **p < .01; ***p < .001; ****p < .0001

To explore the anti‐tumour effects of CD44v6 CAR‐T cells within the body. Luc^+^ MV4‐11 and Luc^+^ SKM‐1 cells (1 × 10^7^) were respectively inoculated into the left groin of BALB/c‐nu mice by subcutaneous injection on day 0. Luc^+^ MV4‐11 mice got the treatment of 1 × 10^7^ CD44v6 CAR‐T cells, NT cells or 200 μl PBS on days 8 and 15, and tumour burden was analysed by BLI on days 7, 14, 21 and 28 (Figure 4A). Comparatively to NT cells or PBS treatment, CD44v6 CAR‐T treatment prevented tumour progression in Luc^+^ MV4‐11 mice (Figure 4B,C). Luc^+^ SKM‐1 mice were negative controls as wild‐type SKM‐1 cells lack CD44v6 expression. The results showed that CD44v6 CAR‐T cells failed to protect Luc^+^ SKM‐1 mice. (Figure 4D–F).

We established a xenogeneic model of AML in NOD/Shi‐scid IL2rγnull (NOG) mice to verify the anti‐leukemic capacity of CD44v6 CAR‐T against constructed *FLT3* mutated AML cells in vivo. NOG mice were intravenously injected with 1 × 10^7^ Luc^+^ SKM‐1 or Luc^+^ SKM‐1‐FLT3 cells on day 0, and treated with of 1 × 10^7^ CD44v6 CAR‐T cells, NT cells or 200 μl PBS on days 15 and 29. The leukaemia development was monitored by BLI on days 14, 28 and 42(Figure 4G). In Luc^+^ SKM‐1‐FLT3 mice, CD44v6 CAR‐T cells prevented leukemic progression (Figure 4H,I), which were not found in Luc^+^ SKM‐1 mice (Figure 4J,K).

### 3.6 Genetic mutations promoted the expression of CD44v6

**FIGURE 5:**
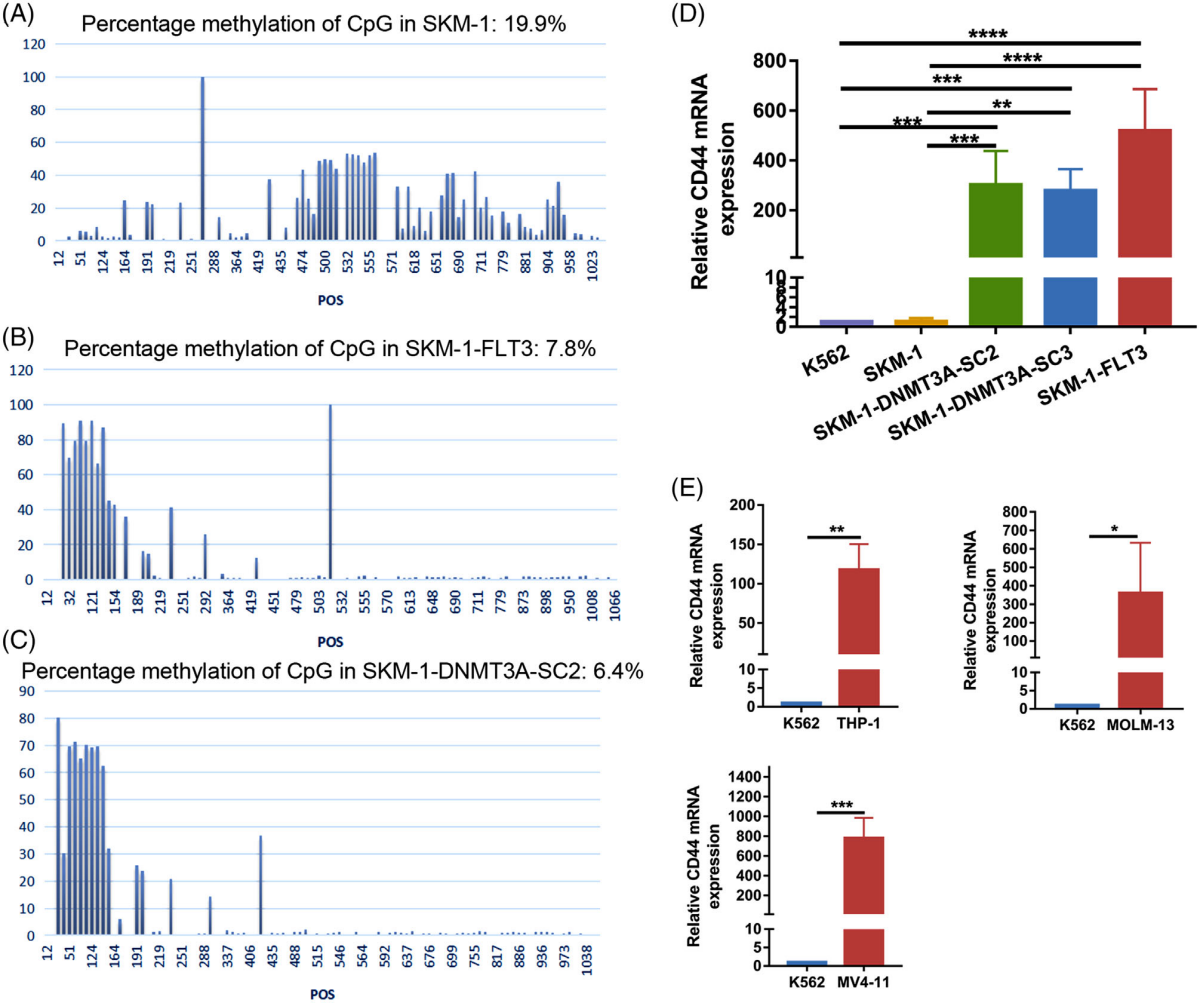
Genetic mutations promoted the expression of CD44v6 in AML cells. Quantitative DNA methylation assays for each CpG site in the CD44 promoter in (A) SKM‐1 cells, (B) SKM‐1‐FLT3 cells and (C) SKM‐1‐DNMT3A‐SC2 cells by EpiTyper. (D) RT‐qPCR analysis of the expression of CD44 in K562 (*n* = 5), SKM‐1(*n* = 5), SKM‐1‐DNMT3A‐SC2 (*n* = 5), SKM‐1‐DNMT3A‐SC3 (*n* = 5), SKM‐1‐FLT3 (*n* = 4). (E) RT‐qPCR analysis of the expression of CD44 in K562 cells (*n* = 4) and AML cell lines THP‐1 (*n* = 3), MOLM‐13 (*n* = 4), MV4‐11 (*n* = 4). Bar are depicted as the mean ± SD. *p < .05; **p < .01

To explore whether *FLT3* or *DNMT3A* mutations affect other molecules of AML, we examined CD33 and CD123 expressions of SKM‐1, SKM‐1‐FLT3 and SKM‐1‐DNMT3A‐SC2 cells. The findings indicated that *FLT3* mutations promoted the expression of CD33 in SKM‐1‐FLT3 cells, while *DNMT3A* mutations did not affect the expression of CD33 and CD123 in SKM‐1‐DNMT3A‐SC2 cells (Figure S3). Our data demonstrated that both *FLT3* and *DNMT3A* mutations promoted the expression of CD44v6 in AML cell lines. How do they affect CD44v6 expression? Since *DNMT3A* is closely associated with the regulation of DNA methylation, we quantitatively evaluated the methylation levels of the CD44 promoter in SKM‐1, FLT3 or DNMT3A mutant SKM‐1 cells through mass spectrometric analysis of DNA methylation (EpiTyper).^29^ We demonstrated that the CD44 promoter methylation was 19.9% in SKM‐1 cells, 7.8% in SKM‐1‐FLT3 cells and 6.4% in SKM‐1‐DNMT3A‐SC2 cells, respectively (Figure 5A–C). We also found high expression of CD44 mRNA in SKM‐1‐FLT3, SKM‐1‐DNMT3A‐SC2, SKM‐1‐DNMT3A‐SC3 (Figure 5D), THP‐1, MOLM‐13, MV4‐11 cells (Figure 5E) and low expression in K562 and SKM‐1 cells, which is consistent with CD44v6 expression in these cells.

### 3.7 Safety of CD44v6 CAR‐T cells

**FIGURE 6:**
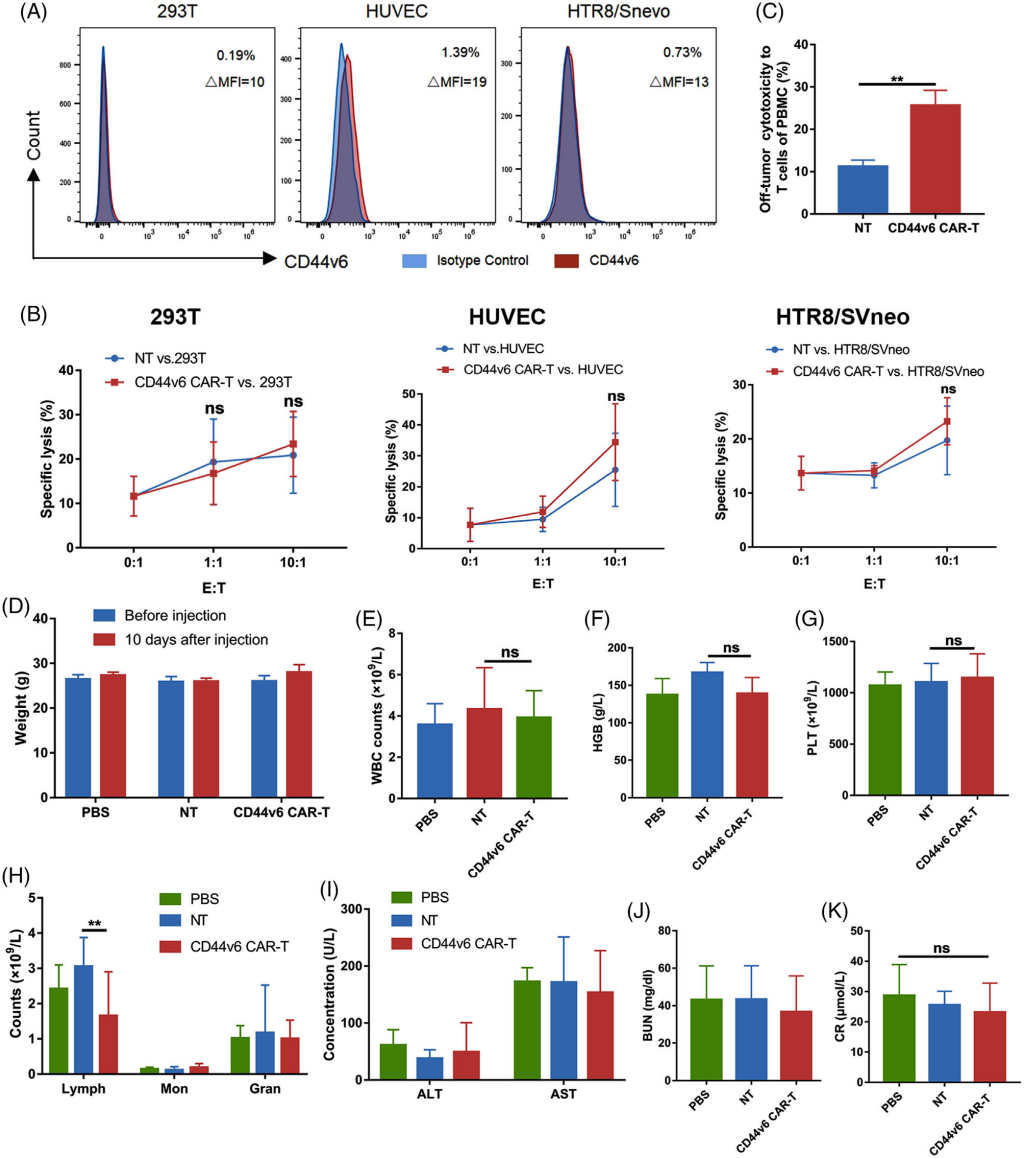
Safety of CD44v6 CAR‐T cells. (A) CD44v6 expression in human normal cell lines 293T,HUVEC and HTR8/Snevo analysed by flow cytometry. (B) CD44v6 CAR‐T cells lysed human normal cell lines 293T (*n* = 4), HUVEC (*n* = 7) and HTR8/Snevo (*n* = 4) at the indicated E:T ratio of 0:1, 1:1 and 10:1 for 24 h. NT cells were used to evaluate unspecific lysis. (C) PBMCs of healthy donors (*n* = 6) were labelled with CFSE and co‐cultured with CD44v6 CAR‐T orNT cells at an E:T of 10:1 for 24 h, and the percentage of dead CD3^+^ T cells of PBMCs were detected by flow cytometry using Fvs and anti‐CD3. (C–J) BALB/c mice were intravenously injected with 1 × 10^7^ CD44v6 CAR‐T cells (*n* = 5), 1 × 10^7^ NT cells (*n* = 5), and 200 μl PBS (*n* = 5) to assess the off‐target toxicity of CAR‐T cells. After 10 days, the weight change of the mice was detected (D), and peripheral blood was collected for blood count analysis (E–H) and liver and kidney functions (I–K). Bar are depicted as the mean ± SD. (E) The WBC; (F) the HGB; (G) the PLT; (H) the subsets of WBC; (I) the ALT and AST; (J) the BUN; (K) the CR. HUVEC, human umbilical vein endothelial cell; PBMC, peripheral blood mononuclear cell. **p < .01; ns not significant

We used human embryonic kidney 293T cells, human trophoblastic HTR8/SVneo cells and human umbilical vein endothelial cells (HUVECs) as target cells to evaluate the safety of CD44v6 CAR‐T cells. Because they did not express, or express very low levels of CD44v6 (Figure 6A). Compared with NT cells, CD44v6 CAR‐T cells had no specificity towards 293T and HTR8/SVneo cells, while CD44v6 CAR‐T cells showed slight cytotoxicity towards HUVEC cells, but without statistical significance (Figure 6B). The slight off‐tumour cytotoxicity to T lymphocytes was detected (Figure 6C). In addition, the result was confirmed in vivo. Amino acid homology between human CD44 antigen isoform 6 precursor (NP_001189484.1) and mouse CD44 antigen isoform f precursor (NP_001171258.1 ) was investigated using NCBI protein BLAST program. BLAST database search showed that the mouse CD44v6 exhibited the high alignment score (569), low *E‐value* (0), and shared similar identity (68%) with human CD44v6 (Figure S4). The alignment score is the total alignment score or the best local alignment score calculated by BLAST when two protein sequences are compared. *E value* < 0.05 represents statistical significance, and the lower the *E value*, the higher confidence of the similarity between two protein sequences.^30^ The *E value* of the similarity between human CD44V6 and mouse CD44V6 is 0, indicating that human CD44V6 and mouse CD44v6 are homologous. Therefore, we evaluated the safety of CD44v6 CAR‐T cells in BALB/c mice. BALB/c mice were injected with 1 × 10^7^ CD44v6 CAR‐T cells, NT cells or 200 μl PBS via tail. The weight of the mice did not change after 10 days of injection (Figure 6D). Compared with the PBS and NT groups, the WBCs, HGB and PLTs in CD44v6 CAR‐T group were not affected (Figure 6E–G). Compared with the NT group, lymphocytes in CD44v6 CAR‐T group were decreased, but still within normal range (Figure 6H). The liver and kidney functions of the CD44v6 CAR‐T group were not affected (Figure 6I–K). CD44v6 CAR‐T cells injections did not result in any serious side effects.

## 4 DISCUSSION

At present, the main factors restricting the CAR‐T treatment in AML are the selection of specific antigens and complex intrinsic cytogenetic or molecular genetic abnormalities. Bypassing the molecular abnormalities themselves and carrying out immunotherapy intervention according to their characteristic immunophenotype is an important model of precision treatment. Few studies have focused on the connection of molecular abnormalities and antigen selection in AML.^31^, ^32^ Here, we confirmed that CD44v6 was highly expressed in *FLT3* or *DNMT3A* mutant AML cell lines or primary AML cells, whereas it was absent in normal HSCs, proving that CD44v6 was an ideal target for AML patients with *FLT3* and *DNMT3A* mutations. Indeed, CD44v6 CAR‐T cells displayed a strong anti‐leukemic effect against CD44v6^+^ AML cells, especially AML cells with *FLT3* or *DNMT3A* mutations.

Studies have reported that CD44v6 is abundantly expressed in various malignant tumours, including breast, gastrointestinal, hepatocellular and colorectal cancers, as well as AML^33^, ^34^, ^35^, ^36^, ^37^. Besides, CD44v6 expression directly correlates with tumour progression.^17^ We found that CD44v6 was abnormally overexpressed in *FLT3*/ITD^+^ AML cell lines and primary AML cells. For AML cell lines with FLT3 mutation (MOLM‐13 and MV4‐11) or FLT3 overexpression (THP‐1), higher level of CD44v6 on their cell surface was also observed. To prove that the CD44v6 expression in AML may be associated with *FLT3* and other genetic mutations, we constructed SKM‐1‐FLT3 cells with *FLT3*/ITD mutation and SKM‐1‐DNMT3A‐SC2, SKM‐1‐DNMT3A‐SC3 cells with *DNMT3A* R882H mutation using SKM‐1 cells, respectively. As expected, CD44v6 expression was increased in all cells engineered to obtain *FLT3* and *DNMT3A* mutations. It has been reported that CD33 and CD123 are classic markers of AML for immunotherapies.^10^, ^11^ Our results showed that *FLT3* mutation promoted the expression of CD33, but not CD123 in SKM‐1‐FLT3 cells, while *DNMT3A* mutation had no effect on the expression of CD33 and CD123. These data indicated that the connection with *FLT3* or *DNMT3A* and CD44v6 might be specific. Moreover, no or little CD44v6 expression was detected on normal hematopoietic cells including bone marrow stem/progenitor cells.^18^, ^20^ As a result, we consider CD44v6 an ideal target for AML patients with *FLT3* or *DNMT3A* mutations.

After verifying the expression of CD44v6 in AML, we constructed CD44v6 CAR‐T cells. The early proliferation of CD44v6 CAR‐T cells was slow as CD44v6 expression on T cells led to transient fratricide. The subsequent proliferation of CD44v6 CAR‐T cells was accelerated which may be associated with downregulated CD44v6 expression and reduced CD8^+^ cytotoxic T cells.^38^, ^39^ Although CD44v6 CAR‐T cells exhibited slight off‐tumour toxicity against T cells, we found that they have no specific cytotoxicity against HSCs and normal cells such as HUVECs, 293T and HTR8/SVneo cells in vitro. Because mouse CD44v6 was homologous to human CD44v6, we investigated the safety of CD44v6 CAR‐T cells in BALB/c mice. Only a slight lymphopenia was noticed with CD44v6 CAR‐T cells injections. Casucci et al. found that CD44v6 CAR‐T produced monocytopenia in vivo, but this was mild and reversible, demonstrating that CD44v6 CAR‐T was safe.^18^

Furthermore, we demonstrated specific cytotoxicity of CD44v6 CAR‐T cells against AML cells in vitro and in vivo. Compared to the CD44v6 negative K562 cells, AML cell lines with *FLT3* mutation (MOLM‐13 and MV4‐11) or FLT3 overexpression (THP‐1), which expressed higher level of CD44v6, were significantly lysed by the CD44v6 CAR‐T. Similar results could be observed in our *FLT3*/ITD and *DNMT3A* mutant SKM‐1 cells in vitro. The above killing process was accompanied by obvious T cell activation and typical cytokine secretion. IFN‐γ was the most significant among them, and other cytokines such as interleukin‐6 and TNF‐α also increase concomitantly. For primary AML leukaemia blast with *FLT3*/ITD mutation, CD44v6 CAR‐T presented significant anti‐leukaemia effects but no obvious killing to the healthy donor, indicating the safety of normal myeloid cells. In animal issue, we proved that CD44v6 CAR‐T cells were an efficacious therapy for AML with *FLT3* mutations in cell derived xenograft (CDX) animal models with the *FLT3* mutated MV4‐11 and *FLT3*/ITD knock‐in SKM‐1 cells. These findings confirmed that CD44v6 CAR‐T cells eliminated CD44v6^+^ AML cells, especially AML cells with *FLT3* or *DNMT3A* mutations.

The next concern was why CD44v6 overexpressed in AML cells with *FLT3* or *DNMT3A* mutations? We quantitatively detected the methylation of the CD44 promoter in SKM‐1, *FLT3* or *DNMT3A* mutated SKM‐1 cells, and found the methylation of CD44 promoter in SKM‐1 cells was the highest. Consistent with our study, hypermethylation of CD44 promoter also promoted downregulation of CD44v6 expression in liver cancer and colorectal cancer.^40^, ^41^ Studies indicated that promoter region CpG methylation could make gene silent. This was also confirmed by our results that the methylation of CD44 promoter was highest and the expression of CD44 mRNA was lowest in SKM‐1 cells compared with SKM‐1‐FLT3 and SKM‐1‐DNMT3A‐SC2 cells. These findings suggested that *FLT3*/ITD and *DNMT3A* mutations decreased CpG methylation in the promoter region of CD44 in SKM‐1‐FLT3 and SKM‐1‐DNMT3A‐SC2 cells, thereby promoting the expression of CD44 and CD44v6.

In conclusion, we demonstrated that CD44v6 CAR‐T cells showed potent anti‐leukemic efficacy and safety in vitro and in vivo. In addition, we revealed that *FLT3* or *DNMT3A* mutations induced CD44v6 overexpression by downregulating the methylation of CD44 promoter. Thus, the CD44v6 CAR‐T therapy may prove to be an efficacious option for AML, especially with *FLT3* or *DNMT3A* mutations.

## CONFLICT OF INTEREST

The authors declare no conflict of interest.

## Supporting information

Supporting Information

## Data Availability

The data that support the findings of this study are available from the correspondings author upon reasonable request.
