# Supplementary material for: CD44v6 chimeric antigen receptor T cell specificity towards AML with FLT3 or DNMT3A mutations
Source: Clin Transl Med. 2022 Sep 26;12(9):e1043. doi: 10.1002/ctm2.1043 (PMC9513046; doi:10.1002/ctm2.1043)
Supplement: Supplementary file 1 — Supporting Information [file CTM2-12-e1043-s001.docx]

Supplementary Materials for

**CD44v6 chimeric antigen receptor T cell specificity towards AML with FLT3 or DNMT3A mutations**

Ling Tang^1†^, Hongming Huang^2†^, Yutong Tang^1†^, Qing Li^3^, Jue Wang^4^, Dengju Li^4^, Zhaodong Zhong^1^, Ping Zou^1^, Yong You^1^, Yang Cao^4^, Yingjie Kong^1^, Anyuan Guo^5^, Shu Zhou^6^, Huimin Li^4^, Fankai Meng^4*^, Yi Xiao^4*^，Xiaojian Zhu^4*^

Correspondence to: [zhuxiaojian@hust.edu.cn](mailto:zhuxiaojian@hust.edu.cn)

Materials and Methods

**AML and healthy donor samples**

Following informed consent from healthy donors and untreated AML patients, we collected peripheral blood according to a protocol approved by the Institutional Review Board of Tongji Medical College and the Hubei committee.

**Cell lines and cell culture**

Human colorectal cancer cell lines (SW480, Caco-2, HT29 and HCT116), human AML cell lines (THP-1, MOLM-13, MV4-11), human chronic myelogenous leukaemia (CML) cell line K562, human embryonic kidney cell line 293T, human umbilical vein endothelial cell line HUVEC, human trophoblastic cell line HTR8/SVneo, human lung cancer cell line A549 and human hepatocellular carcinoma cell line HepG2 were obtained from the China Center for Type Culture Collection (Wuhan, China). The SW480, Caco-2, HT29, HCT116, 293T, HUVEC, HTR8/SVneo, A549 and HepG2 cells were maintained in Dulbecco’s modified Eagle’s medium (DMEM) supplemented with 10% FBS and 100 U/ml penicillin-streptomycin. All leukemia cell lines were grown in RPMI 1640 with 10% FBS and 100 U/ml penicillin-streptomycin at 37 °C in 5% CO_2_.

**MassArray EpiTYPER quantitative DNA methylation analysis**

Bisulfite conversion of the genomic DNA was performed with the EZ DNA Methylation-Gold™ Kit, Zymo Research Corporation (Irvine, CA, USA) according to the manufacturer’s protocol. Quantitative DNA methylation analysis was performed with MassArray EpiTyper (Sequenom, San Diego, CA, USA). The promoter regions of the *CD44* gene in SKM-1, SKM-1-FLT3 and SKM-1-DNMT3A-SC2 cells were analyzed. The *CD44* gene sequence and primers are shown in Supplemental file: Materials and Methods. The quantitative methylation data for each CpG site or aggregates of multiple CpG sites obtained from MassArray were analyzed on the EpiTYPER software (Sequenom).

The CD44 gene sequence :

CD44 chr11 :35160280-35161452

**
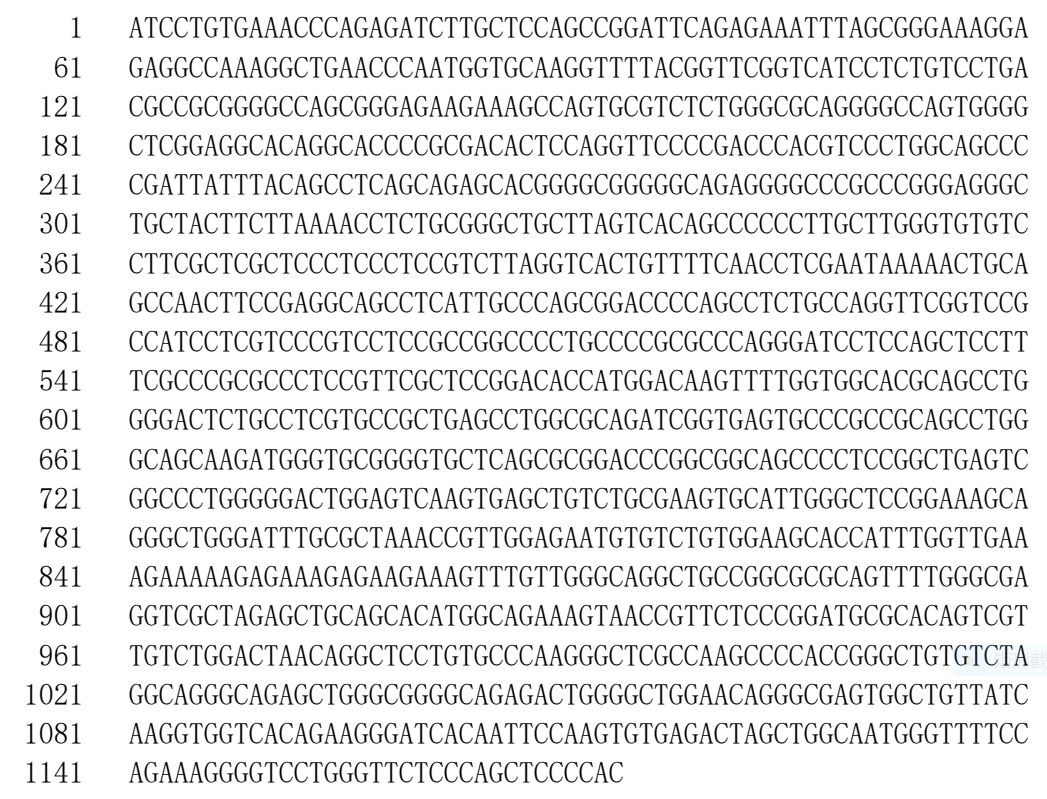
**

The primer sets of methylation analysis in the promoter region of *CD44* gene used in DNA methylation assay：

| **Primer Name** | **Sequence (5′ –3′)** |
| --- | --- |
| CD44_F1 | TCCCTACACGACGCTCTTCCGATCTTTTGTGAAATTTAGAGATTTTGTTT |
| CD44_R1 | AGTTCAGACGTGTGCTCTTCCGATCTAAACTATAAATAATCRAAACTACCAAA |
| CD44_F2 | TCCCTACACGACGCTCTTCCGATCTGAGGTATAGGTATTTYGYGATATTT |
| CD44_R2 | AGTTCAGACGTGTGCTCTTCCGATCTAAACTACCTCRAAAATTAACTACAAT |
| CD44_F3 | TCCCTACACGACGCTCTTCCGATCTATTGTAGTTAATTTTYGAGGTAGTTT |
| CD44_R3 | AGTTCAGACGTGTGCTCTTCCGATCTTACCACCAAAACTTATCCATAATATC |
| CD44_F4 | TCCCTACACGACGCTCTTCCGATCTGATATTATGGATAAGTTTTGGTGGTA |
| CD44_F4 | AGTTCAGACGTGTGCTCTTCCGATCTATACTTCCACAAACACATTCTC |
| CD44_F5 | TCCCTACACGACGCTCTTCCGATCTGAGAATGTGTTTGTGGAAGTAT |
| CD44_R5 | AGTTCAGACGTGTGCTCTTCCGATCTCTATAACCACCTTAATAACAACCACT |

Note: Y=C+T;R=G+A


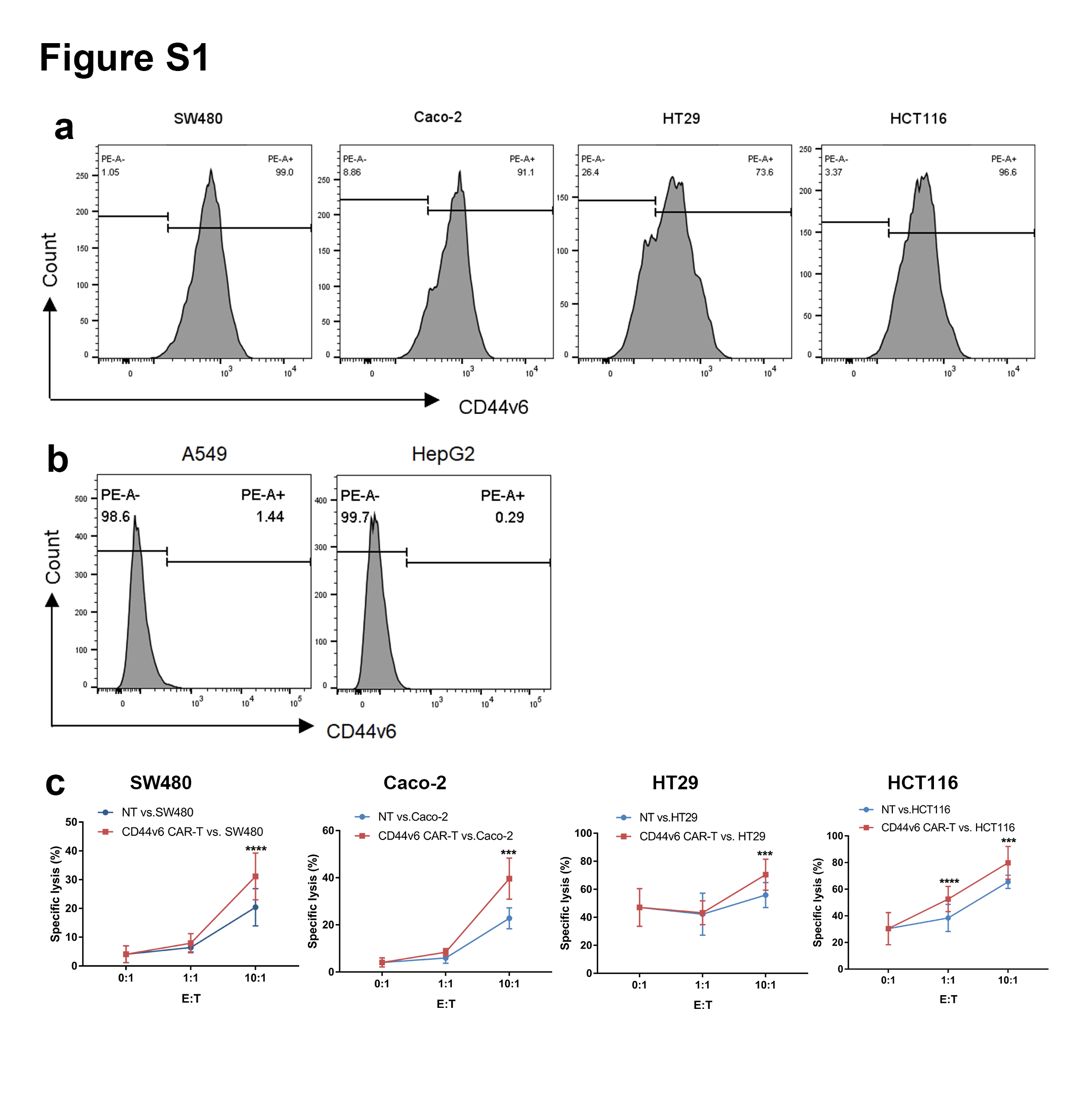


Figure. S1. a. Flow cytometric analysis of CD44v6 expression on colorectal cancer cell lines (SW480, Caco-2, HT29 and HCT116). **b** Flow cytometric analysis of CD44v6 expression on the A549 and HepG2 cell lines. **c** CD44v6 CAR-T cells lysed CD44v6^+^ colorectal cancer cell lines SW480 (n=5), Caco-2 (n=3), HT29 (n=6) and HCT116 (n=8).


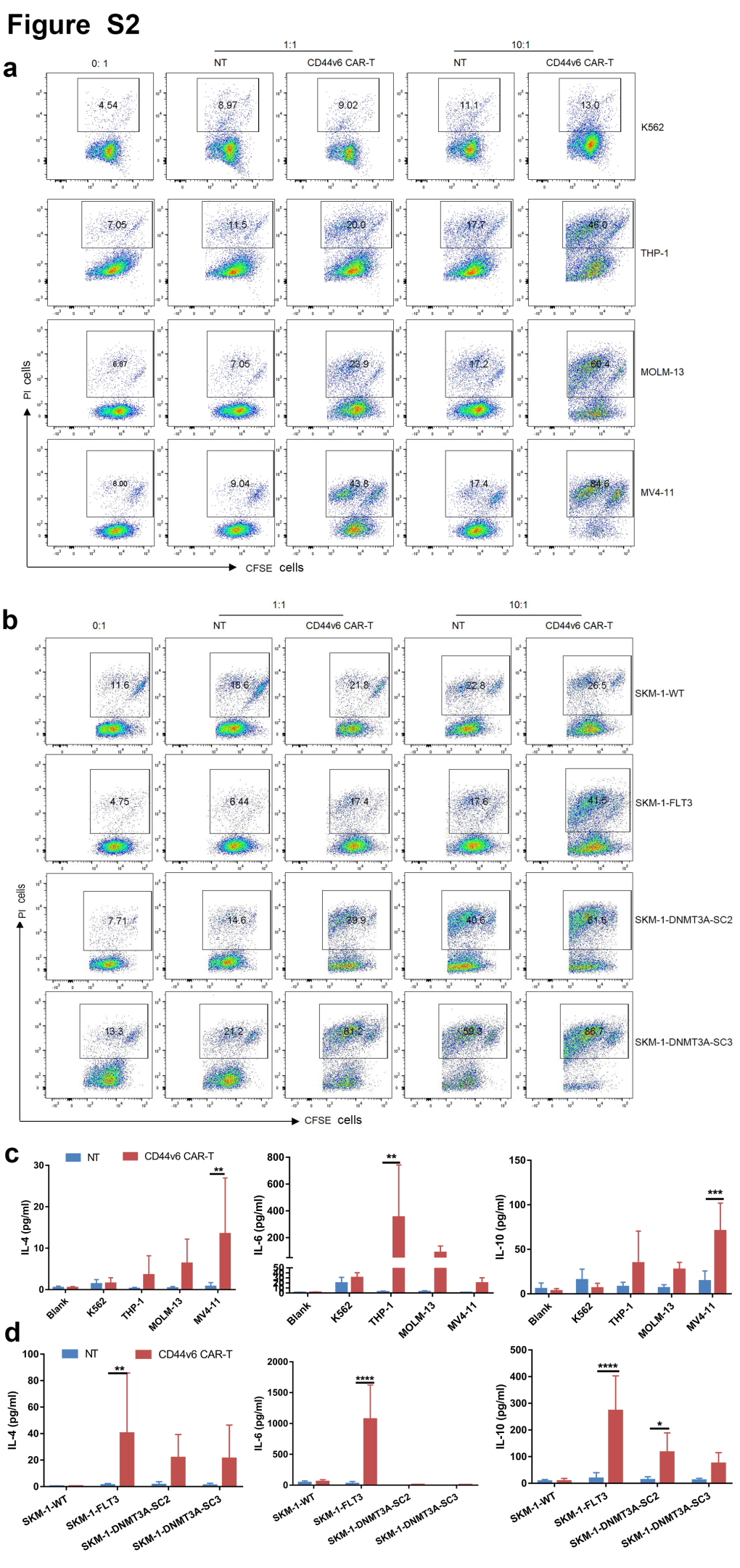


Figure. S2. **CD44v6 CAR-T cells specifically target CD44v6^+^ tumor cell lines**

CFSE-labeled target cells were co-cultured with CD44v6 CAR-T or NT cells at the indicated E:T ratio of 0:1, 1:1 and 10:1 for 24 h. The percentage of live and dead cells was tested by flow cytometry using PI. The CFSE^+^PI^+^/CFSE^+^ ratio was used to determine the killing rate. NT cells were used to evaluate unspecific lysis. **a** representative scatter plot of CD44v6 CAR-T cells lysed K562 cells and CD44v6^+^ AML cell lines THP-1, MOLM-13 and MV4-11. **b** representative scatter plot of CD44v6 CAR-T cells lysed AML cell lines SKM-1, SKM-1-FLT3, SKM-1-DNMT3A-SC2 and SKM-1-DNMT3A-SC3. **c** CD44v6 CAR-T or NT cells (n=4) were co-cultured with K562, THP-1, MOLM-13 and MV4-11 cell lines at an E:T ratio of 1:1 for 24 h. IL-4, IL-6, IL-10 amounts in the supernatants were analyzed by using CBA. **d** CD44v6 CAR-T or NT cells (n=6) were co-cultured with FLT3 or DNMT3A mutant AML cell lines SKM-1-FLT3, SKM-1-DNMT3A-SC2 and SKM-1-DNMT3A-SC3 at an E:T ratio of 1:1 for 24 h. IL-4, IL-6, IL-10 amounts in the supernatants were analyzed by using CBA.

**
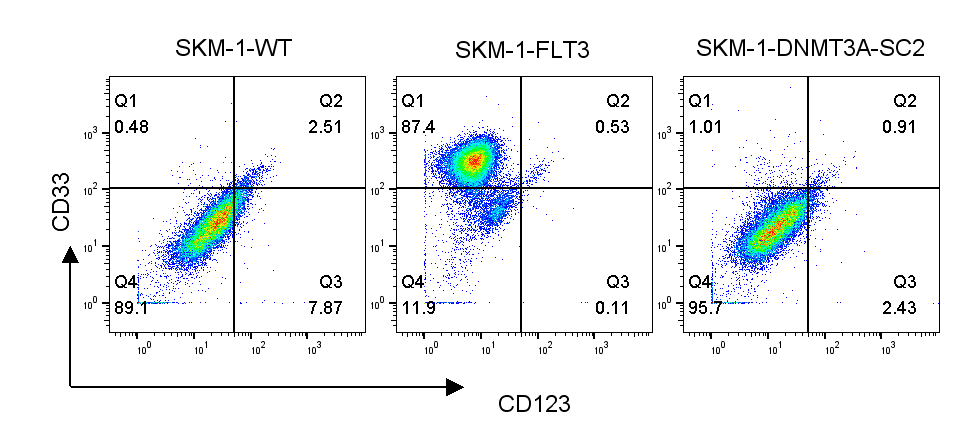
**

**Figure. S3.** Flow cytometric analysis of CD33 and CD123 expression on the SKM-1-WT, SKM-1-FLT3 and SKM-1-DNMT3A-SC2 cell lines.

**
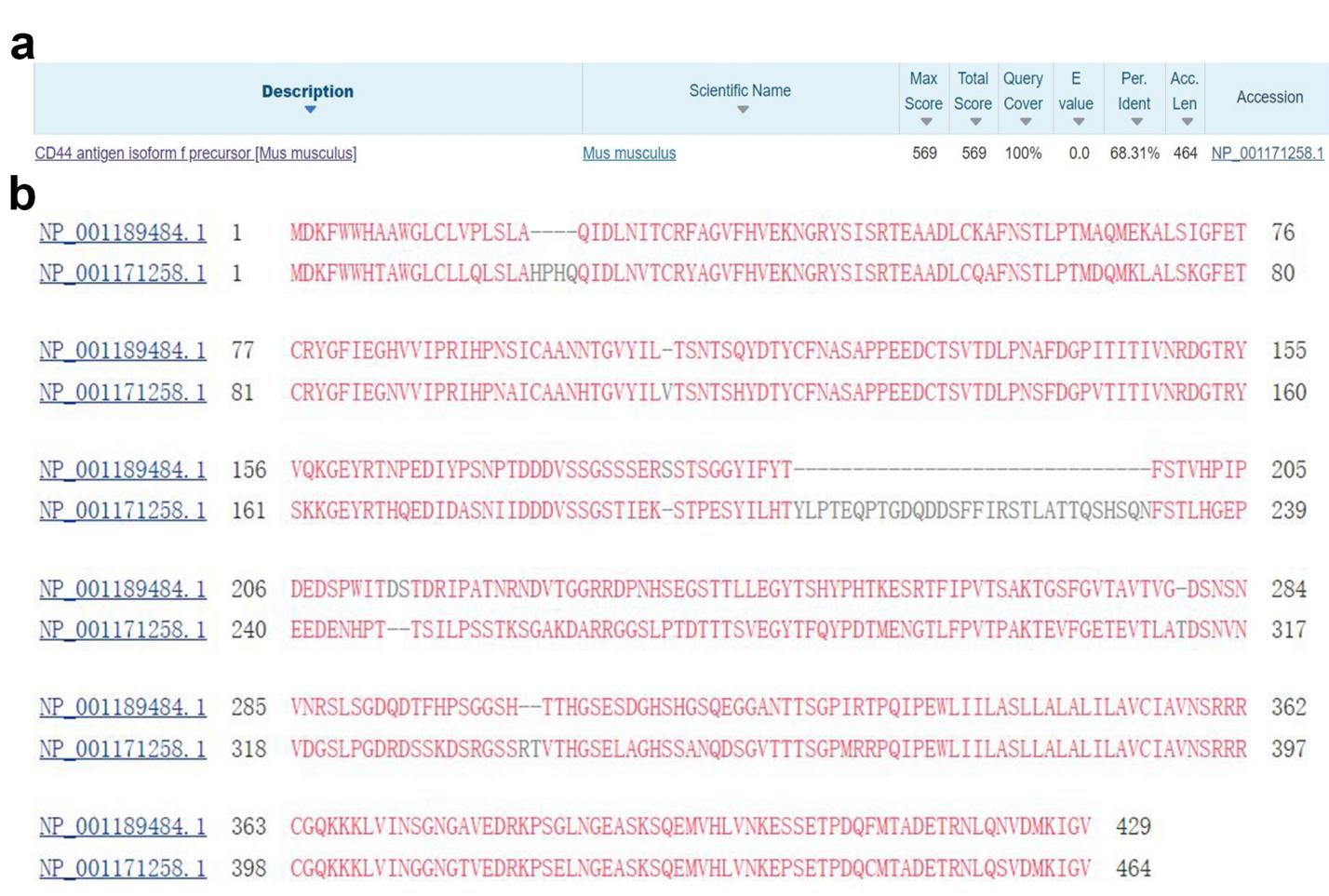
**

**Figure. S4**. Amino acid homology between human CD44v6 and mouse CD44v6 using a similarity search in NCBI protein BLAST program (BLASTP). **a**. Results of amino acid sequence similarity analysis between mouse CD44v6 and human CD44v6. **b** Comparison of mouse and human CD44v6 amino acid sequences.
